# Distant Storms as Drivers of Environmental Change at Pacific Atolls

**DOI:** 10.1371/journal.pone.0087971

**Published:** 2014-01-31

**Authors:** Jonathan P. A. Gardner, David W. Garton, John D. Collen, Daniel Zwartz

**Affiliations:** 1 Centre for Marine Environmental and Economic Research, Victoria University of Wellington, Wellington, New Zealand; 2 School of Biology, Georgia Institute of Technology, Atlanta, Georgia, United States of America; 3 Antarctic Research Centre, Victoria University of Wellington, Wellington, New Zealand; University of Sydney, Australia

## Abstract

The central Pacific Ocean with its many low lying islands and atolls is under threat from sea level rise and increased storm activity. Here, we illustrate how increasing frequency and severity of large scale storm events associated with global climate change may be particularly profound at the local scale for human populations that rely on lagoon systems for provision of a variety of goods and services. In August 2011 a storm originating in the Southern Ocean caused a large amplitude ocean swell to move northward through the Pacific Ocean. Its arrival at Palmyra Atoll coincided with transient elevated sea surface height and triggered turnover of the lagoon water column. This storm-induced change to the lagoon reflects long distance connectivity with propagated wave energy from the Southern Ocean and illustrates the increasing threats generated by climate change that are faced by human populations on most low-lying Pacific islands and atolls.

## Introduction

It has been suggested that the modern era be known as the ‘anthropocene’ because human activity has had, and is increasingly having, a significant influence on the global environment [1], [2], with low-lying and densely populated Pacific atolls and islands being particularly vulnerable to such changes [3], [4], [5], [6]. Over the coming years the world will experience a period of environmental challenges, arising chiefly from increased anthropogenically-induced global climate change [7], [8], [9]. Increasingly, parts of the world will experience modified weather patterns [10], new storm tracks [11], increased frequency of major storm activity, and new weather extremes [6], [12], [13], [14], [15]. The social, economic and political consequences arising from such changes are profound, and are predicted to include impacts on native ecosystems, hydrology and water resources, coastal systems, tourism, human settlements and infrastructure, and human health [3], [6], [16], [17], [18].

Storm event and sea swell signals generated in one region have been recorded thousands of kilometers away [19], [20]. Whilst environmental impacts at distant sites may not be significant because energy is dissipated over the long distance that the signal has travelled, event intensity in one region can be sufficient to cause significant environmental disturbance at a distant site [20]. With new storm tracks and with storm activity and weather instability predicted to increase over the next century [11], [14], our observations at a low lying Pacific Ocean atoll demonstrate that the spatial scale of environmental impact resulting from global climate change may be greater than previously realised.

**Figure 1:**
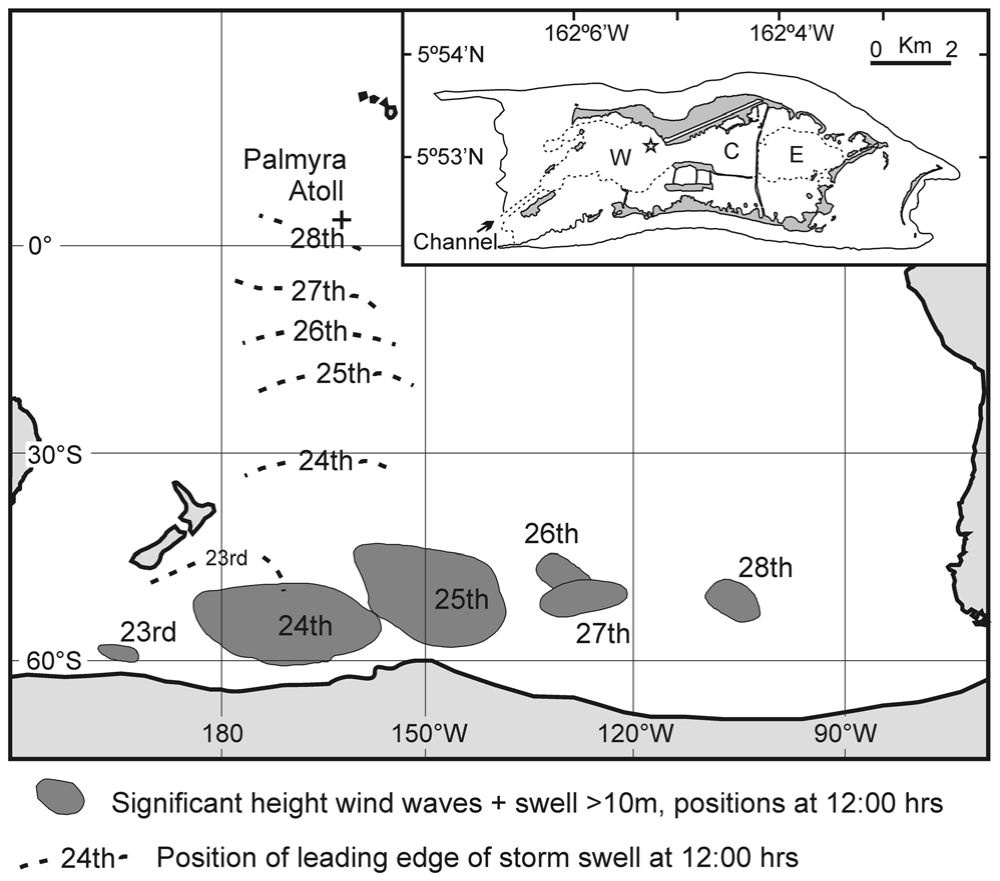
Track of major Southern Ocean storm event, late August 2011 (data derived from Husson et al. 2012). Shaded regions represent the centre of the storm and associated region with elevated sea surface height (>10 m). Dashed lines indicate the progress of the leading edge of increased swell height propagated northwards by this event. Inset: Palmyra Atoll with three lagoon basins (West, Center and East lagoons indicated by W, C and E, respectively). Reference site in West Lagoon for profiles presented in Figs. 2 and 3 is indicated by the star symbol).

Palmyra Atoll (Palmyra – Figure 1, inset) is a remote collection of islets located in the central Pacific Ocean, 1700 km southwest of Hawaii and near the northern end of the Line Islands at 05°52′N, 162°05′W [21], [22]. Whilst the atoll has no permanent human inhabitants, it is representative of many inhabited Pacific atolls. Palmyra's lagoon system is composed of three lagoons (maximum depth of 52 m) that are partially connected to the open ocean via small, shallow cuts between islets and by the main Entrance Channel that is up to 12 m deep [23], [24], [25]. Multi-year study of the lagoon system at Palmyra has revealed water column stratification in the absence of a strong pycnocline [25]. Lagoon surface waters are oxygen-saturated but bottom waters are sulphidic and anoxic, with high H_2_S and elevated ammonia concentrations, and decreased pH, temperature and chlorophyll *a* concentrations. It is uncertain how typical this water column structure is of atoll lagoons in general, given the paucity of relevant studies, but the water column at Clipperton Island is also known to be dysoxic/anoxic in its deeper regions [25], [26], [27], Niau lagoon (French Polynesia) has an unstudied anoxic layer [27], and dysoxia develops at some sites in Penryhn Atoll lagoon (Cook Islands) (*pers. obs.*).

During late August 2011, swells originating from a particularly large storm centre in the Southern Ocean [28] passed across Palmyra (Figure 1) whilst we were working there. Despite the magnitude of the storm event, little data are available to describe it in detail. The storm originated to the south of New Zealand in the Southern Ocean (near Antarctica) and moved along an easterly track, until it dissipated off the southwestern coast of South America. The storm generated waves that radiated from this intense and short-lived event, which, consistent with other such reports, was poorly observed [28]. While a storm event of this magnitude must have affected a large number of Pacific islands and atolls, we have no data to confirm this, and only one anecdotal report of increased and out of phase tides in parts of French Polynesia. This “once in a decade” storm event (as it was described in Hawai'i once it reached there) provided us with a unique opportunity to assess the local impact of a very large scale storm on the long term structure and stability of the atoll's lagoon system. We were able to achieve this because we had been studying this particular lagoon for several years prior to the storm and had a “baseline” data set about the pre-disturbance structure and apparent stability of the lagoon system. Not only did the storm event cause localised damage to the atoll's islets, it also resulted in the complete turnover of the water masses in the lagoon system. This is the first time that such a lagoon turnover event has been recorded. We highlight how future increased storm events may cause more lagoon turn events with unexpected consequences for human populations that are dependent on tropical lagoon systems for many goods and services.

## Materials and Methods

We surveyed water column structure in Palmyra's three lagoons at a series of stations for the period 2007–2009 [25]. Further data for many of these sites were added in June 2010, August 2011 (during the storm event), June and August 2012, and June 2013. Casts were made using two RBR CTD sensors fitted with dissolved oxygen probes (fast response OxyGuard Ocean sensor, DO522M18) and turbidisensors (Seapoint Ltd). In this study, four variables were measured: dissolved oxygen (%DO - per cent of saturation; actual values varied from zero to ∼250 µmol L^−1^), temperature (°C), salinity (PSU - converted from conductivity) and depth (m - converted from pressure). Density was derived from the logger software. During 2011 and 2012 we used a hydrogen sulphide (H_2_S) probe (AMT H2S 48M memory probe with an amperometric micro-sensor with ≤200 milliseconds response time; recording frequency once per second) to measure dissolved H_2_S in bottom waters. This probe also measured pH with a sensitivity of ±0.01 pH units. The H_2_S probe and the CTD were strapped together during casts to ensure direct depth-dependent comparability of measurements. All sites were located by handheld GPS. CTD casts were conducted at <1 m s^−1^, with the CTDs recording every second. To avoid background noise caused by moving water at depths ≥20 m, joint CTD and H_2_S probe casts were lowered at 0.25 to 1 m increments, allowing sufficient time at each depth interval for the H_2_S sensor to stabilise. Signals derived from movement of the H_2_S probe were removed and the values at each stable level were averaged (n = 700±300) and used for plotting data. Tide predictions for Palmyra Atoll [29] were referenced to the predictions in Hawaii. The water level inside the lagoon was recorded by a Sutron RLR tide gauge operated by the University of Hawaii Sea Level Center. The offset of tide gauge datum from the NOAA tidal datum was estimated by taking the average over one year's observations. The water level anomaly was obtained by subtracting the interpolated predictions from the tide gauge observations.

Research at Palmyra Atoll was conducted under authority of permits issued to JPAG by the US FWS. All necessary permits were obtained for the described study, which complied with all relevant regulations. We thank the University of Hawaii Sea Level Center for permission to use their sea level data. Data files used in this study have been deposited for public access with the Palmyra Atoll Research Consortium - http://www.palmyraresearch.org/

## Results

**Figure 2:**
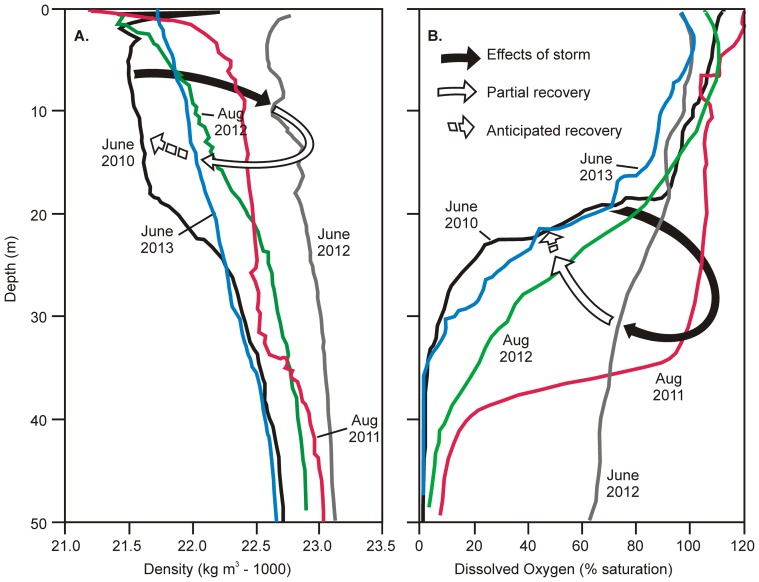
Water column profiles West Lagoon, Palmyra Atoll, June 2010 through August 2012. 2A: Typical density stratification (black, June 2010) disrupted and progressing to uniform, higher density (red, Aug 2011; green June 2012) during turnover and reverting towards stratified condition (yellow, Aug 2012; blue, June 2013). 2B: Shallow oxycline of June 2010 disrupted by storm event (Aug 2011) followed by return of oxycline in August 2012 and June 2013 (line colours same as for 2A). Solid arrows indicate temporal progression of gradients; dashed line predicted recovery towards conditions typical for Palmyra Atoll. Note: For clarity data only for West Lagoon are presented; nearly identical patterns occur independently in the other two lagoons at Palmyra Atoll.

**Figure 3:**
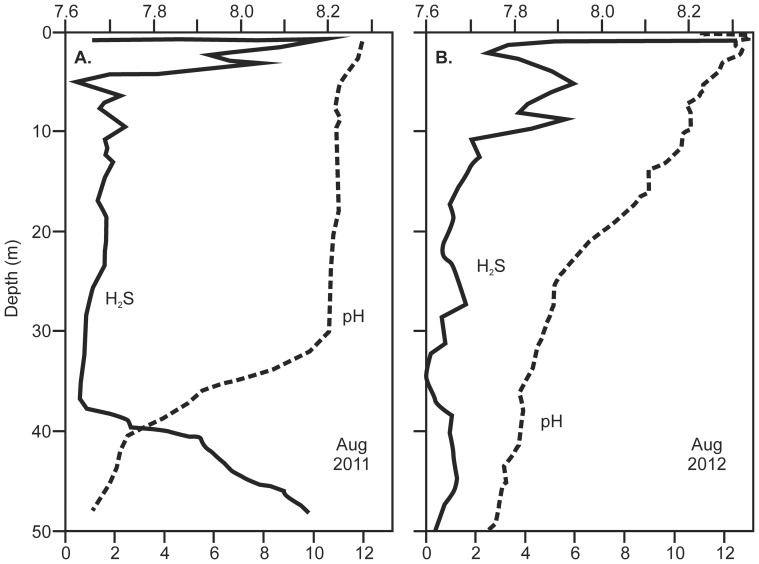
Water column profiles West Lagoon, Palmyra Atoll, for H_2_S (solid line) and pH (dashed line). 3A: August 2011 - anoxic conditions with both H_2_S and pH showing strong gradients associated with dissolved oxygen (see Figure 2B). 3B: August 2012 - Absence of oxycline following storm-driven turnover removes gradient in H_2_S and pH which had not fully recovered by August 2012 although decline in pH indicates recovery is in progress. Note: The amphoteric H_2_S sensor is sensitive to water currents and wave-induced motion, thus elevated recordings from shallow depths (less than 10 m) are spurious (H_2_S cannot persist in oxic water).

**Figure 4:**
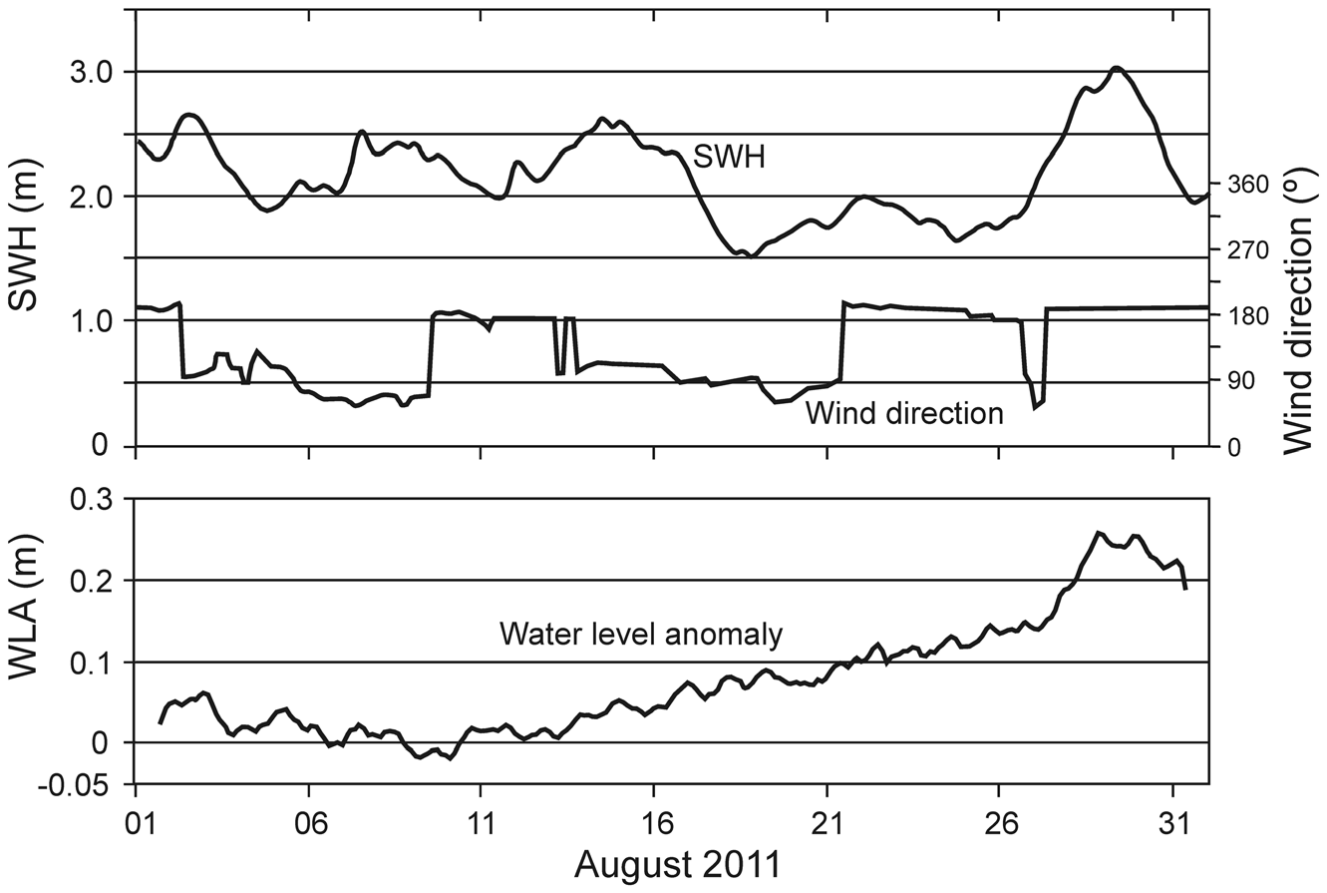
Significant wave heights (SWH), wind directions and water level anomaly (WLA) records at Palmyra Atoll, August 2011. The modest increase in tidal height was associated with neap tides and strong winds from the southwest that persisted sufficiently to trigger turnover in the weakly stratified lagoon system.

Pre-storm data [25], in conjunction with records made at the time of the storm (August 2011) and subsequently (June and August 2012, June 2013), demonstrate the consequences of long distance connectivity of energy from the storm event: first, a modest increase in sea surface height associated with the storm event disrupted directional surface flow and caused complete water column turnover in the lagoon system, and second, the lagoon water column returned rapidly to its pre-disturbance state. Before the storm event of August 2011 water column structure in all three lagoons was characterised by pronounced stratification, with oxygenated surface waters and sulphidic/anoxic water below ∼25 m depth (Figs. 2 and 3). During the August 2011 storm event the oxycline was depressed by ∼20 m, reflecting the influx of oxygen-saturated oceanic seawater into the lagoon system as the storm-generated swell and elevated SSH coincided at Palmyra. Sampling ten months later (June 2012) revealed persistence of the storm-induced disturbance (Figure 2B), with the absence of an oxycline, the first such occurrence in six years of observation, and no evidence of anoxic bottom water at 50 m depth. However, in August 2012, 12 months after the storm event, and only two months after the June 2012 measurements, there was evidence of the oxycline re-establishing at ∼22 m depth (Figure 2B). This pattern of recovery to the pre-storm structure was further confirmed in June 2013. The re-established oxycline reflected concurrent changes in the physical stratification of the lagoon water column towards a pre-storm density gradient profile (Figure 2A). The density gradient typical of pre-storm conditions was shifted to a uniform and higher density as oceanic seawater entered the lagoon system and mixed throughout the water column (Figure 2A), but by June 2013 the density gradient was returning to its pre-disturbance state. The H_2_S and pH profiles parallel the patterns observed in DO throughout this two year period (Figure 3). Prior to the storm event (June 2010) H_2_S in the anoxic layer (below 30 m depth) peaked at approximately 10 mg L^−1^ and pH declined from 8.3 at the surface to 7.6 at the bottom. Post-storm mixing disturbed this pattern, which then shows incomplete recovery through August 2012. Radar altimetry data from ERS/Envisat (DEOS, Technical University of Delft, The Netherlands) show that a transient sea surface height anomaly of about +0.2 m moved past Palmyra in late August 2011. The NOAA Wavewatch 3 global hindcast [30] indicates that the swell from the Southern Ocean storm, with a significant wave height of 3 m, also arrived there between 28–30 August (Figure 4). The period of maximum water level anomaly values coincided with a spring tide and strong southwesterly winds that also contributed to sustained high water levels in the lagoon system. This set of combined circumstances is very different from anything recorded by us during the previous six years, despite coincident spring tides and southwesterly winds.

## Discussion

Our results indicate that full turnover of lagoon water column structure of Pacific atolls can occur in association with large and powerful storm events in the Southern Ocean or, by extension, in the North Pacific Ocean. Our ability to detect the effects at an equatorial site of a large storm originating in the Southern Ocean (or the North Pacific Ocean) underscores the importance of long-distance connectivity between ocean regions, which will increase in significance if extreme weather events also increase in frequency.

Palmyra is now characterised by a series of islands and islets, the channels between which are deepening and widening as a consequence of natural processes and the breakdown of the solid land rim that was constructed in the 1940s [23], [24], [25]. Pronounced wave action from the south, generated by the Southern Ocean storm event, washed over the top of some islets on the atoll's southern shore, cut one island into two thereby creating a new channel between the lagoon and the ocean on the southern shore, and coincident with large high tides flowed into the lagoon system through the many channels between islets and islands that now exist all along the coastline (both north and south shore) of the atoll. The extensive shallow area (typically <2 m deep at high tide) to the east of Palmyra that is largely isolated from the main lagoon system (i.e., from West, Center and East lagoons) also experienced elevated sea levels, resulting from the input of oceanic water via large waves overtopping the low-lying islets and islands and also entering the system via the channels between the islets/islands. Thus, all areas of the lagoon system at Palmyra were affected.

Turnover of stratified lagoons is associated with potential hazards for surrounding coral reef systems that provide a variety of goods and services to human populations (e.g., act as a buffer against direct wave action, provide a source of building material, act as a source of food items such as reef fish, provide an important source of revenue in terms of tourism). H_2_S is highly toxic to most forms of metazoan life, yet during this single event the release of H_2_S-saturated bottom water (∼10 mg L^−1^) from the lagoon basins onto the extensive coral reef systems surrounding Palmyra Atoll appears to have occurred without detectable negative impacts. We suggest that the absence of impact is largely due to the fact that wind, mostly from the northeast, mixed the surface waters of the lagoon and also dispersed the gas before it could build up to a concentration sufficient to be detected and/or impact the atoll's biota. It is presently not known how many lagoon systems are characterised by deep, anoxic (H_2_S-laden) waters simply because the physico-chemical properties of atoll lagoon systems remain unstudied in any detail. Palmyra's lagoon is thought to be H_2_S-laden in its deeper regions as a consequence of human-mediated modifications of the atoll in the early 1940s [23], [24], [25]. Given that most inhabited atolls have been substantially modified by activities such as filling in natural channels between islets, dredging and dumping, and cutting of large channels for shipping, it is likely that many lagoons have been converted from well-flushed to a static, stratified state, with associated build-up of dysoxic or even anoxic conditions in deeper waters. Thus, lagoon turnover events as described at Palmyra are likely to have occurred at other Pacific atolls, but unless coupled with observable events such as fish kills, have largely gone unrecognised and unstudied. Anecdotally, we are aware of reports from Penrhyn Atoll (Cook Islands) of deepwater turnover events in the lagoon that result in many fish being described as being “sick and lethargic”, often breaching the surface waters, and gasping. Given Penrhyn lagoon's depth and bottom profile, this may be a lagoon turnover event comparable to that detailed by us for Palmyra.

Assessment of the lagoon water column properties, in particular of DO and density, shows that all or very nearly all of the water in the lagoon system at Palmyra was replaced during a brief period by oxygenated, denser oceanic water, despite limited connection between the lagoon system and the surrounding ocean. Lagoon turnover events as observed at Palmyra are previously unreported and highlight the need for continuous monitoring to record infrequent, yet ecologically highly significant, events. The ability to identify the lagoon turnover event at Palmyra is largely a function of the fact that the lagoon system is characterised by pronounced stratification, and secondarily by the fact that Palmyra Atoll is arguably the most intensively studied atoll anywhere in the world. While we cannot know for sure, it seems highly likely that other lagoon systems in the south-central Pacific Ocean region have been similarly affected. However, such events in other locations may not be of the same sort as that experienced at Palmyra because other lagoons may not be as deep as at Palmyra and might may not be strongly stratified, at least not to the same extent (because so few atoll lagoons have been studied in detail this latter point must remain speculative). Nonetheless, a storm event of this magnitude will have noticeable local consequences, both in terms of damage to atoll shorelines plus associated reef systems, and also will result in a brief but substantial period of flushing of the lagoon by oceanic water. While this is the first report linking tropical lagoon turnover to disturbance from a storm originating in the Southern Ocean, it seems likely that such events occur on a repetitive basis given the frequency and severity of major storm events [19], [31]. Predictions of increasing storm frequency and severity as a consequence of global climate change [4], [12], [13], [14], [15] suggest the potential for future turnover events to also occur with greater frequency. Indeed, there is evidence of intensification of storm events in the Pacific Ocean during the last decade [31].

The present report confirms the connectivity between Southern Ocean storms and tropical Pacific regions with associated changes in lagoon circulation dynamics, allowing dysoxic and H_2_S-rich water to be released into the surrounding environment. Whilst the coral reef environments of many tropical islands and atolls are considered to be the most vulnerable ecosystem to temperature change [3], we highlight the additional threat posed by distant storms as drivers of environmental change at Pacific atolls. The environmental and social consequences of lagoon system turnover at tropical atolls remain unclear, but turnover events at atolls with human populations, which place additional pressure on the lagoon for goods and services, has the potential for generating greater impacts on surrounding sensitive coral reef systems and on the atoll's human inhabitants.
